# Lipid Deposition and Metabolism in Local and Modern Pig Breeds: A Review

**DOI:** 10.3390/ani10030424

**Published:** 2020-03-03

**Authors:** Klavdija Poklukar, Marjeta Čandek-Potokar, Nina Batorek Lukač, Urška Tomažin, Martin Škrlep

**Affiliations:** 1 Agricultural Institute of Slovenia, Ljubljana SI-1000, Slovenia; klavdija.poklukar@kis.si (K.P.); meta.candek-potokar@kis.si (M.Č.-P.); nina.batorek@kis.si (N.B.L.); urska.tomazin@kis.si (U.T.); 2 University of Maribor, Faculty of Agriculture and Life Sciences, Hoče SI-2311, Slovenia

**Keywords:** pig, adipose tissue, local breeds, modern breeds, cellularity, transcriptome, proteome, adaptation

## Abstract

**Simple Summary:**

Intensive selective breeding and genetic improvement of relatively few pig breeds led to the abandonment of many low productive local pig breeds. However, local pig breeds are more highly adapted to their specific environmental conditions and feeding resources, and therefore present a valuable genetic resource. They are able to deposit more fat and have a distinct lipogenic capacity, along with a better fatty acid composition than modern breeds. Physiological, biochemical and genetic mechanisms responsible for the differences between fatty and lean breeds are still not fully clarified. The present paper highlights important associations to better understand the underlying mechanisms of lipid deposition in subcutaneous and intramuscular fat between fatty and lean breeds.

**Abstract:**

Modern pig breeds, which have been genetically improved to achieve fast growth and a lean meat deposition, differ from local pig breeds with respect to fat deposition, fat specific metabolic characteristics and various other properties. The present review aimed to elucidate the mechanisms underlying the differences between fatty local and modern lean pig breeds in adipose tissue deposition and lipid metabolism, taking into consideration morphological, cellular, biochemical, transcriptomic and proteomic perspectives. Compared to modern breeds, local pig breeds accumulate larger amounts of fat, which generally contains more monounsaturated and saturated fatty acids; they exhibit a higher adipocyte size and higher activity of lipogenic enzymes. Studies using transcriptomic and proteomic approaches highlighted several processes like immune response, fatty-acid turn-over, oxidoreductase activity, mitochondrial function, etc. which differ between local and modern pig breeds.

## 1. Introduction

The domestic pig (*Sus scrofa*) originated from wild boar through multiple independent domestications approximately 9000 years ago [1,2,3]. Throughout the ages, a large variety of local pig populations were independently selected for morphological and behavioural traits and were well adapted to the environmental conditions in which they were raised [4]. From the second half of the 20^th^ century onwards, pig production has been characterized by intensive selective breeding and genetic improvement of relatively few breeds, while many local pig breeds have been abandoned due to low productivity [4,5,6]. Nowadays local pig breeds are generally reared in extensive farming systems and are well adapted to specific environmental conditions and feeding resources [6]. Local breeds are considered to have better meat quality, enabling the production of high-quality meat products [7,8]. In modern pigs, long-term selection and breeding programs have resulted in enhanced ability for reproduction and growth, higher carcass leanness, more efficient muscle accretion [9], reduced fatness, including intramuscular fat (IMF) content, and reduced fat quality, which is important for processing aptitude and sensory properties of meat products [10]. Local pig breeds are more highly adapted to their local environment conditions, like shortages in food availability and repetitive seasonal cycles of fasting. After a period of food shortages, they are able to accumulate large amounts of body fat when food is more readily available [11]. Local breeds also exhibit a lower potential for growth and protein deposition than modern pig breeds [12,13] and are therefore less attractive to breeders. However, the awareness of the importance of biodiversity and the adverse side effects of intensive production systems in the last few decades has led to the increased interest in the breeding of local pig breeds [14]. Growth performance of twenty European local pig breeds has recently been reviewed [15] and the study demonstrated that there is a lack of knowledge about their growth potential and their nutritional requirements that could otherwise be used to optimize the management of these breeds. The efficiency and quality of pig meat production depend to a large extent on the metabolic processes involved in producing and depositing lipids. In order to optimize production traits in pigs, it is therefore important to better understand the biological processes involved in the determinism of lipid metabolism in adipose tissue of pigs. 

Adipose tissue is the largest and metabolically very dynamic energy reservoir. There, most of the energy reserves are stored as triglycerides, which are incorporated into fat cells, also known as adipocytes. Fat tissue in the body can be deposited as visceral, subcutaneous, intermuscular (between the muscles), or intramuscular (within the muscle) depots with decreasing accretion intensity during development [16]. Each fat depot shows specific metabolic properties [17] which in return influence the whole-body metabolism by secreting hormones, adipocytokines and regulatory proteins. Fat tissue-derived hormones affect a range of processes like nutritional intake, inflammatory response or sensitivity to insulin [18]. Hormonal regulation is also breed-dependent and associated with fat synthesis (lipogenesis) and breakdown (lipolysis and fatty acid oxidation) [19]. Molecular mechanisms responsible for the differences between the fatty phenotypes of local pig breeds and the lean phenotypes of modern pig breeds are still not fully elucidated. The objective of the present article was to review the physiological and biochemical characteristics, differentially expressed genes and proteins, contributing to a better understanding of mechanisms responsible for differences in fat deposition between distinct metabolic types of pig breeds.

## 2. Lipogenic Potential and Fatty Acids Composition in Local and Modern Pig Breeds

### 2.1. Subcutaneous Adipose Tissue

#### 2.1.1. Fatty Acids Composition of Subcutaneous Adipose Tissue

**Figure 1:**
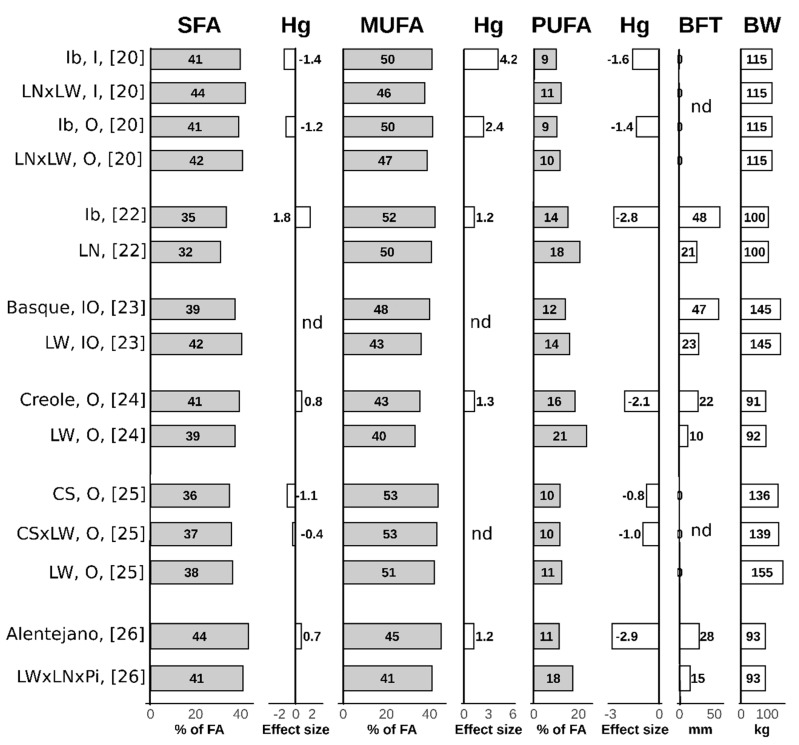
Fatty acid composition of subcutaneous adipose tissue of local vs. modern pig breeds and within-study effect size (Hedges’ g) [27]. Studies were carried out in different conditions (including body weight, backfat thickness) and should be interpreted accordingly. Effect size (Hedges’g) [27] is considered to be medium if it is above 0.5 or below −0.5 and large if it is above 0.8 or below −0.8. In the case of Reference [20], the effect size for the inner and outer backfat layer was calculated separately. SFA = saturated fatty acids; MUFA = monounsaturated fatty acids; PUFA = polyunsaturated fatty acids; Hg = Hedges’ g; BFT = backfat thickness; BW = body weight; I = inner back fat layer; O = outer back fat layer; FA = fatty acids composition; Ib = Iberian pig breed, LN = Landrace; LW = Large White; Pi = Piétrain; CS = Cinta Senese; nd = calculation of Hedges’g was not possible due to missing data.

In modern pig breeds, the goals of selective breeding resulted in a strongly reduced lipogenic potential, while local pig breeds preserved this capacity along with a distinctive fat metabolism and fatty acids composition [20], in particular for monounsaturated fatty acids (MUFA). Besides the effect of the genotype, fatty acids composition is strongly affected by dietary fatty acid intake, and also by production system, age of animals at slaughter, sex, body fat mass and environmental temperature [21]. Figure 1 illustrates a summary of published studies that compared modern and local pig breeds with regard to fatty acid composition. A comparison of individual studies is difficult due to different rearing conditions, sampling locations and body weights. For the present review only studies were considered where local and modern pig breeds or cross-breeds were reared in the same environmental conditions, in particular feeding. Figure 1 summarizes the results of these studies [20,22,23,24,25,26] along with Hedges’ g effect size calculation [27] based on the data reported. Along with higher adiposity, a greater saturated fatty acids (SFA) content is a characteristic of local pig breeds. In addition, subcutaneous adipose tissue of local pig breeds contains a higher proportion of MUFA, mainly oleic acid, and a lower proportion of polyunsaturated fatty acids (PUFA), in both cases with a larger effect size (i.e., 1.2-4.2 for MUFA and 0.8-2.9 for PUFA) than in the adipose tissue of modern pigs [20,22,23,24,25,26]. Since PUFAs are obtained directly from the ingested feed [28] and MUFAs could be deposited either from feed or by desaturation of saturated fatty acids (SFA) (obtained by de novo SFA synthesis) [21], a higher MUFA content is implicated in a higher ability of local pig breeds to synthetize and desaturate fat. Simultaneously, an increase in MUFA and SFA lowers PUFA content due to the effect of dilution. 

#### 2.1.2. Lipogenic Enzyme Activities of Subcutaneous Adipose Tissue

Contrary to other livestock species, in pigs the adipose tissue is a primary site of lipid synthesis, i.e., lipogenesis [29]. Lipogenesis is defined as the conversion of glucose into triglycerides [30] and it provides at least 80% of the deposited fatty acids in pigs [31]. Fatty acids biosynthesis depends on the activity of several key enzymes (Table 1), like fatty acid synthase (FAS), malic enzyme (ME), glucose-6-phosphate dehydrogenase (G6PDH) and acetyl-CoA carboxylase (ACACA) (Table 1) [32], and it occurs under the regulation of the physiological stage of animals (i.e., age, weight), their genetic predisposition and their sex [33,34].

The literature comparing the lipogenic enzyme activity of local and modern pig breeds is scarce. However, the available reports of the studies (Table 2) demonstrate important breed differences in lipogenic enzyme activities. 

Along with high lipid synthesis capacity, local breeds exhibit higher lipogenic enzyme activities than modern pig breeds, as illustrated by the higher activities of lipogenic enzymes ACACA, G6PDH, ME and FAS in Alentejano vs. Large White, and in Iberian vs. Landrace x Large White breeds [34,35]. On the other hand, the activities of FAS and ME were found to be lower in local Basque pigs than in Large White pigs, slaughtered at 320 and 228 days, respectively, which also showed the important effect of the pigs’ maturity [23]. The studies reporting the activity of SCD also prove the importance of the stage of maturity. For instance, lower activity of SCD in fatty Meishan pigs compared to modern Large White pigs was not in accordance with a slightly higher content of monounsaturated oleic acid in the backfat of Meishan pigs, suggesting a higher desaturation capacity in local pig breeds during earlier development [36]. Stearoyl-CoA-desaturase is under regulation of numerous factors, which either stimulate (i.e., insulin, carbohydrates) or inhibit (i.e., leptin, PUFA, state of hunger) its expression [37], and its activity reflects the interaction of genetic and environmental factors. Local pig breeds are considered to have a more persistent ability of desaturation during periods of fasting, which was shown in the case of Iberian compared to Duroc pig breeds. Inhibition of the SCD after 24 h of fasting was more intense in Duroc pigs compared to Iberian pigs [38]. 

### 2.2. Intramuscular Fat

Intramuscular fat consists of phospholipids, triglycerides and cholesterol. The balance between synthesis, degradation and uptake of triglycerides is reflected in IMF content. Intramuscular triglycerides are mainly stored in adipocytes but also as droplets in the myofiber cytoplasm [39]. Intramuscular fat content is highly correlated with the sensory acceptability of pork [40,41,42], and with several other traits like water holding capacity [24] and tenderness [41]. The flavor and juiciness of pork are enhanced when the IMF content is higher than 2.5% [42]. Breeding of modern pig breeds resulted in a lower IMF deposition [43] together with a higher carcass lean meat percentage and lower backfat thickness [44]. Intramuscular fat content is related to muscle structure and composition [45]. In addition, it is positively associated with oxidative metabolic type [46] and backfat thickness [47,48]. Local pig breeds generally exhibit more oxidative muscle metabolism and a higher IMF. For instance, in *longissimus dorsi*, a predominantly glycolytic type of muscle, a higher IMF along with a higher percentage of oxidative muscle fibres was found in local Pulawska than in modern Polish Large White pigs [49], as well as a higher IMF content along with the higher expression of *longissimus dorsi* MyHC I isoforms in Korean native black pig, compared to in the Landrace breed [50]. 

#### 2.2.1. Fatty Acids Composition of Intramuscular Fat

**Figure 2:**
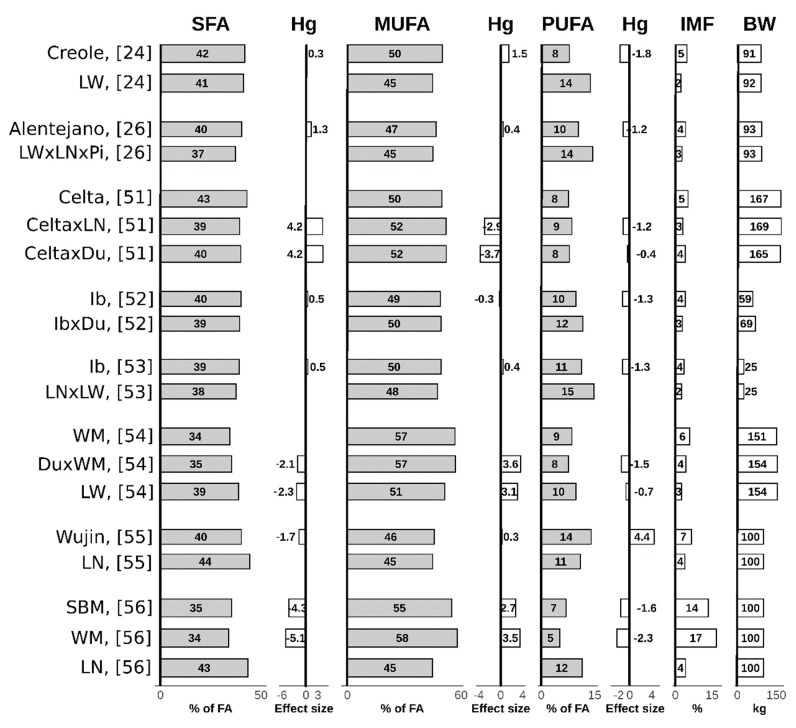
Fatty acids composition of intramuscular fat in *longissimus dorsi* muscle at given body weights of local vs. modern pig breeds or cross-breeds and within a study effect size (Hedges’ g) [27]. Studies differ with respect to experimental conditions (including BW and IMF) and should be interpreted accordingly. The effect size (Hedges’ g) [27] is considered to be medium if it is above 0.5 or below −0.5 and large if it is above 0.8 or below −0.8. SFA = saturated fatty acids; MUFA = monounsaturated fatty acids; PUFA = polyunsaturated fatty acids; Hg = Hedges’g; IMF = intramuscular fat; BW = body weight; LW = Large White; LN = Landrace; Pi = Piétrain; Ib = Iberian; Du = Duroc; WM = White Mangalitsa; SBM = Swallow bellied Mangalitsa.

In agreement with a higher genetic capacity to deposit IMF, local breeds generally exhibit an increased proportion of SFAs and MUFAs, along with decreased PUFAs proportions. In comparison to modern pig breeds, the differences were in (in regard to the effect size) small to large (i.e., 0.3–5.1), as was demonstrated for Iberian, Creole, White Mangalitsa, Swallow-bellied Mangalitsa, Alentejano and Wujin local pig breeds (Figure 2) [24,26,51,52,53,54,55,56]. In accordance with our assumptions set for subcutaneous fat (i.e., a higher de novo synthesis and desaturation ability), most of the local pig breeds exhibited higher MUFA and lower PUFA contents compared to local pig breeds. Crossbreeding of local pig breeds with modern pig breeds also affected the IMF fatty acids composition, although the effect may be breed-dependant. The proportion of MUFA was higher in crossbreeds than in local and modern pig breeds during the crossing of Celta with the modern breed Landrace, which significantly affected the proportion of MUFA (effect size = 2.9), especially for oleic acid. Similarly, crossing the Duroc breed with Celta also affected the proportion of MUFA (effect size = 3.7) [51]. However, crossing Mangalitsa with Duroc had no significant effect on SFA and MUFA proportions in *longissimus dorsi* muscle [54].

#### 2.2.2. Lipogenic and Lipolytic Enzyme Activities of Intramuscular Fat

In agreement with a higher muscle fat deposition (i.e., IMF), higher lipogenic enzyme activities and lower lipolytic enzyme activities are generally characteristic for local rather than modern pig breeds. Several studies have been conducted to evaluate the lipogenic potential for muscle fatty acid synthesis between local and modern pig breeds [35,55,57,58,59], showing elevated lipogenic and desaturation capacity and decreased lipolysis in local compared to modern pig breeds (Table 3).

A close positive relationship between muscle malic enzyme activity and IMF deposition was reported, both of which were higher in local breeds [35,58]. Higher lipogenic capacity was also indicated by the activities of ACACA, ME and G6PDH in *semimembranosus* muscle, which was found to be significantly higher in local Basque compared to in the modern Large White breed [57]. Similarly, the activity of the same enzymes in *semimembranosus* muscle was higher in local Meishan compared to in the Large White breed [58]. Likewise, FAS activity was higher in the *longissimus dorsi* muscle of local Wujin compared to in Landrace pig breed, along with higher Δ-9 desaturation activity, resulting in a higher MUFA deposition [55]. The same study also indicated lower hormone-sensitive lipase (HSL) activity in Wujin, similar to for the comparison of local Mashen and Large White breeds [59], demonstrating a lower capacity for lipid mobilization. It can, therefore, be assumed that lipogenesis is elevated and lipolysis is restrained in fatty local breeds compared to in lean modern pig breeds.

### 2.3. Summary of Lipogenic Potential and Fatty Acids Composition Differences in Local and Modern Pig Breeds

Local pig breeds preserved a high capacity of lipid deposition, which is reflected in an increased amount of subcutaneous adipose tissue and IMF content compared to genetically improved modern pig breeds. With elevated fatness in local breeds, lipogenic and desaturation enzyme activities are increased in the early stages of pig’s maturity compared to modern breeds, while lipolytic enzyme activity is reduced. In accordance, the fatty acids composition of subcutaneous adipose tissue and IMF content demonstrated a higher MUFA content in local breeds than in modern pig breeds, also indicating the higher ability of local breeds to synthetize and desaturate fat.

## 3. Adipose Tissue Cellularity and Biochemical Processes 

Adipose tissue growth is a consequence of hypertrophy (increase in size of adipocytes) and hyperplasia (increase in the number of adipocytes). The changes are induced by imbalance in lipid metabolism, which is caused by an increased efflux of free fatty acids into adipose [60]. Adipocytes increase their number and size with animals’ weight and age, thus affecting backfat thickness and IMF content [57,61]. In the early stages of life, a pig’s adipose tissue grows mainly due to hyperplasia, the process characterized by proliferation and differentiation of multipotent mesenchymal stem cells into mature adipocytes [62,63]. After the significant increase in the cell number, adipocytes start to magnify (hypertrophy) owing to the accumulation of triglycerides. The increase in the size of adipocytes is limited and the rate of hyperplasia is increased when their size reaches the maximum. Adipocytes are initially small cells, spherical in shape, possessing high activity in terms of lipid synthesis [62]. After the differentiation, most of their growth is due to lipid accumulation (hypertrophy) through circulating triglycerides or de novo lipogenesis.

Processes of lipogenesis and lipolysis in adipocytes are controlled by several hormones, like insulin, glucagon and catecholamines [60,64,65]. Adipocytes themselves produce and release leptin, which is involved in the regulation of feed intake with inhibition of lipid synthesis and promotion of lipid mobilization [66]. Increased levels of leptin are present in the animals with greater a fat deposition and adipocyte size [67]. Lack of leptin signaling due to the mutation in leptin or the leptin receptor gene or alterations in leptin transport affect the neuroendocrine and immune system, resulting in a stimulated feed intake and reduced energy expenditure [68,69]. Moreover, due to the condition referred to as leptin resistance, even high levels of circulating leptin are not always effective [67]. These phenomena are evidenced in some fatty individuals and are also demonstrated in fatty Iberian pigs [70,71].

Fatty local breeds generally have larger adipocytes diameter than leaner breeds at the same body weight. For example, pigs of fatty Meishan breed were shown to have larger adipocytes than Landrace pigs, in both terms of the inner and outer backfat layers [61]. Moreover, it has been shown that fatty pig breeds exhibited earlier and greater subcutaneous adipose tissue development, as demonstrated in Basque vs. Large White [72] or Meishan vs. Large White pigs [73].

Differences in IMF content between fatty and lean breeds can also be partly explained by the divergent rate of hyperplasia and hypertrophy. One example is the study of Zhao et al. [55], where fatty Wujin pigs were found to have a higher intramuscular adipocyte diameter than Landrace pigs.

To summarize, local pig breeds exhibit larger adipocytes and earlier maturation of adipose tissue than modern pig breeds on a cellular level.

## 4. Transcriptomic Regulation in Fatty and Lean Pig Breeds

### 4.1. Transcriptional Regulation of Adipogenesis

**Figure 3:**
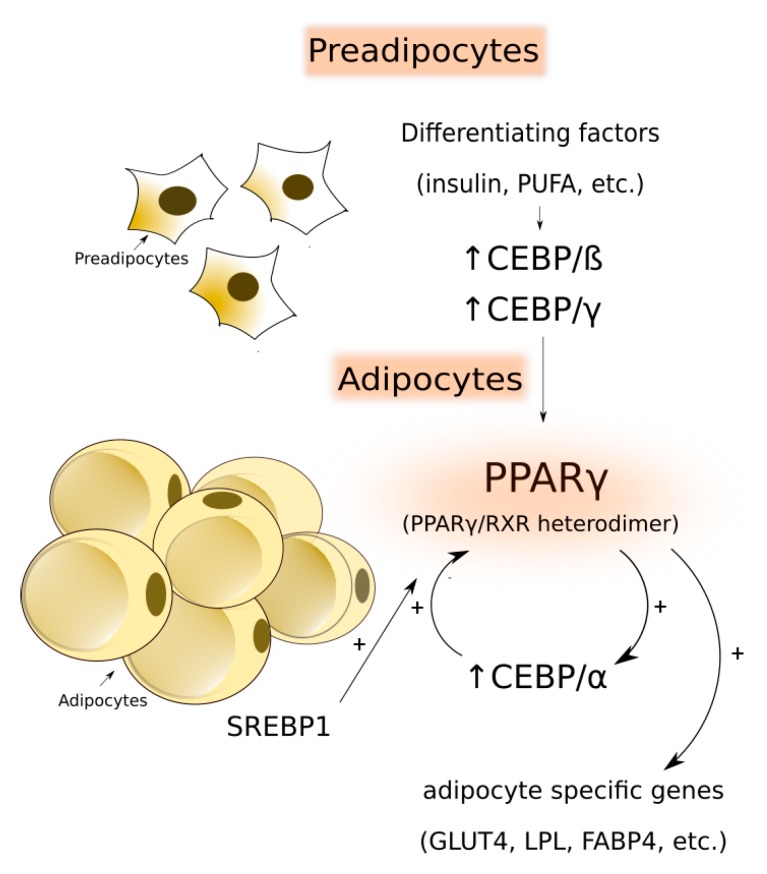
Transcriptional regulation of adipogenesis. ↑ = upregulation of the gene; CEBP/ß = CCAAT/enhancer-binding protein beta; C/EBPγ = CCAAT/enhancer-binding protein gamma; PPARγ = peroxisome proliferator-activated receptor gamma; RXR = retinoid X receptor; C/EBPα = CCAAT/enhancer-binding protein alpha; SREBP = sterol regulatory element-binding proteins; FABP = fatty acid-binding proteins; GLUT4 = glucose transporter type 4, LPL = lipoprotein lipase.

Many review articles described transcriptional regulation of adipogenesis, which is, aside from lipogenesis, crucial for the development of adipose tissue [62,74,75]. A simple diagram of transcriptional regulation behind adipose tissue development is presented in Figure 3. 

Briefly, an increase in the adipocyte number is controlled by many adipogenic stimuli, including insulin, glucocorticoids, dietary PUFA and other differentiating factors, which determine whether adipocytes will start to differentiate or remain quiescent [76]. Initial differentiation starts with a decreased expression of several genes, which is typical for preadipocytes like CCAAT/enhancer-binding proteins (C/EBPß and C/EBPγ), which are activated in response to adipogenic stimuli and induce the expression of the central adipogenic factor peroxisome proliferator-activated receptor gamma (PPARγ) [77,78]. Its signaling pathway promotes fatty acid oxidation, synthesis of triglycerides in adipocytes and causes an increase in adipocyte volumes [62,79]. In the terminal stages of adipogenesis, heterodimer PPARγ induces the expression of another CAAT/enhancer-binding protein - C/EBPα, which is binding on the PPARγ promoter site and positively regulates PPARγ expression [78], and thus ensures maintenance of the differentiated state of the adipocytes [74]. Moreover, sterol regulatory element-binding proteins (SREBP) are also involved in the regulation of PPARγ expression [80]. Some other genes, like lipoprotein lipase (LPL), fatty acid-binding proteins (FABP) or glucose transporter type 4 (GLUT4) bind in their promotor region PPARγ or C/EBPα transcription factor, resulting in activation of the protein translation [62]. 

### 4.2. Transcriptomic Profile in Fatty and Lean Breeds

With the recent development of more advanced methodologies, several transcriptome-based studies comparing fatty and lean pig breeds have been conducted (Table 4). The transcriptomic approach provides an additional tool for the identification of possible molecular mechanisms responsible for variability in fatness between local and modern pig breeds. A high throughput sequencing approach (RNA-seq) has recently become the preferred approach for determination of RNA presence and quantity. Compared to the previously used microarray technique, RNA-seq allows a genome-wide analysis of transcription at the resolution of a single nucleotide, including the detection of different splicing events, post-transcriptional editing events and identification of non-coding RNAs [81]. Although RNA-seq technology is more comprehensive for transcriptome studies, microarrays studies are also valuable for explaining underlying mechanisms of the differences in lipid metabolism between local and modern pig breeds. Despite many advantages, transcriptional profiling presents a challenge in its interpretation due to high tissue heterogeneity. Observed differences in transcriptome profile may be influenced by the cell type composition between samples [82,83]. In subcutaneous adipose tissue, adipocytes are the primary cell type and myofibers represent the primary cell type in skeletal muscle.

#### 4.2.1. Comparison of mRNA Transcriptome of Subcutaneous Adipose Tissue

Underlying differences in distinct fat deposition between fatty local and modern pig breeds could be partly explained by increased lipogenesis in fatty breeds, as was shown in increased expression of adipogenic and lipogenic genes in fatty Bamei and Iberian breeds compared to Large White and Duroc breeds, respectively [38,84]. Excessive fat accumulation is often considered as a state of chronic low-grade inflammation. An inflammatory response could be triggered by several intrinsic signals like adipocyte death, hypoxia or mechanical stress between the cell or extracellular matrix (ECM) caused by extensive tissue remodeling [85]. In a comparative research study where subcutaneous adipose tissue of local Basque and modern Large White pigs was investigated, functional enrichment analysis highlighted the differences in gene groups associated with immune response. The overexpressed genes related to the immune response in Basque pigs could be partly explained by a higher capacity of triglyceride storage in adipocytes and possible necrotic adipocyte death resulting in recruitment of macrophages, which are clearing necrotic debris and enlarging adipose tissue [72]. Genes involved in the inflammatory response were also more highly expressed in the subcutaneous fat of Iberian pigs than in Duroc pigs, indicating low-grade inflammation. Moreover, Iberian pigs exhibited a higher expression of leptin and GLUT4 genes. Leptin is an effective in vitro monocyte chemoattractant, while GLUT4 overexpression might indicate an adaptive response to insulin resistance caused by low-grade inflammation in Iberian pigs [86]. Insulin is stimulating ECM development [87] and the genes involved in ECM were enriched in Duroc than Iberian pigs [86]. In addition, genes involved in insulin signaling pathway and insulin resistance were upregulated in local Songliao pigs compared to in the Landrace pig breed [88]. Functional analysis of differentially expressed genes in the subcutaneous adipose tissue indicated upregulation of lipogenic and adipogenic processes, while processes associated with lipid mobilization and expenditure were downregulated in local breeds compared to modern breeds. A study on several Chinese breeds as compared to Yorkshire breeds revealed upregulated expression of genes associated with immune response, oxidoreductase activity and biosynthetic process (e.g., acetyl-CoA biosynthetic process), whereas the expression of genes involved in fat oxidation was lower [89]. The study of Song et al. [90] showed that genes associated with fatty acid degradation, mitochondrial functions and oxidoreductase activity were downregulated in indigenous Min pigs compared to in modern Landrace pigs. Similarly, genes related to mitochondrial energy and electron transport pathways were also downregulated in local Basque pigs compared to in Large White pigs [72].

#### 4.2.2. Comparison of mRNA Transcriptome of Intramuscular Fat

With regard to intramuscular fat, the expression of genes involved in fatty acids turn-over (i.e., transport, synthesis, degradation) can explain the differences in IMF content between fatty and lean breeds. For instance, functional enrichment analysis revealed a higher expression of genes involved in fatty acid, lipid and phospholipid synthesis in local Basque compared to Large White breed [82] and also in Iberian pigs compared to in Iberian x Duroc crosses [83]. Similarly, a higher expression of genes involved in lipid metabolic process and fatty acid biosynthetic process was demonstrated in Diannan Small-ear pig and Tibetan pig compared to in Landrace and Yorkshire breeds [91], and also in local Jinhua compared to in the Landrace breed [92]. In addition, lower expression of HSL and adipose tissue triglyceride lipase in Wujin breed indicated lower lipolysis, higher lipogenesis and better fatty acid transport compared to Landrace pigs [55]. The same studies [82,83], however, also reported an increased expression of genes for mitochondrial oxidation of fatty acids and lipid degradation, which also indicates higher lipid turnover and a positive association towards oxidative muscle metabolism in local breeds.

A higher *longissimus dorsi* IMF content in local Mashen pigs compared to in modern Large White pigs could be explained by a higher rate of adipogenesis (upregulation of CEBPß, CEBPα and PPARγ in Mashen compared to Large White) [59]. Another comparison of IMF in Wujin and Landrace pigs showed that preadipocytes isolated from muscle tissue of Wujin pigs exhibited a higher adipogenic capacity during the early stages of adipogenesis (e.g., higher expression of PPARγ and CEBPα) and a higher capacity of triglyceride accumulation due to a higher rate of lipogenic genes expression (e.g., higher expression of FASN and SREBP1) in the middle and later stages of adipogenesis than in Landrace pigs [93]. It can be concluded that differences in IMF content between local and modern pig breeds could be ascribed to a higher expression of lipogenic genes and fatty acid transport genes, or to a higher rate of adipogenesis.

### 4.3. Involvement of Non-Coding RNAs in Fat Deposition

In mammals, RNA molecules that lack protein-coding potential (non-coding RNA) are implicated in the regulation of different traits, including fat deposition and lipid metabolism [94]. MicroRNAs (miRNA) are small, typically 19–23 nucleotides long regulatory non-coding RNAs that are decreasing the target mRNA levels or inhibiting the translation of protein-encoding transcripts. Their expression correlates with different biological processes such as development, differentiation and proliferation [95]. In pigs, miRNAs have been demonstrated to be involved in adipogenesis [96], myogenesis [97,98] and lipogenesis [91]. In addition, tissue-specific miRNA expression differed between breeds [91,99]. Moreover, long non-coding RNAs (lnc-RNAs) (transcripts longer than 200 nucleotides) are the second group of non-protein coding transcripts, which have been implicated in the regulation of lipid metabolism and adipogenesis [100,101]. By using RNA-seq, differentially expressed lncRNA and mRNA in subcutaneous adipose tissue of Laiwu and Large White pigs revealed that lncRNAs were mainly involved in the PPAR signaling pathway, while biological processes were associated with fatty acid metabolism and adipocyte differentiation [102]. As for lncRNA implications in IMF development, the differential expression profile of lncRNAs in Jinhua and Landrace pigs also indicated that its involvement in signaling pathways is closely related to lipid metabolism (for instance the mitogen-activated protein kinase signaling pathway) [103].

### 4.4. Summary of Transcriptomic Regulation Differences in Fatty and Lean Breeds

Comparative transcriptomic studies between local and modern breeds enabled us to identify functional networks of genes that can affect the fatty phenotype in local breeds. In local compared to modern breeds, studies revealed upregulation of genes involved in adipogenesis, lipogenesis, immune response and insulin signaling/resistance and downregulation of genes involved in extracellular matrix formation and mitochondrial energy metabolism in the subcutaneous adipose tissue. In the case of IMF, fatty local breeds demonstrated a higher expression of genes implicated in adipogenesis, lipogenesis but also fatty acid mobilization and expenditure, which corroborates with the higher level of oxidative metabolism characteristic of local breeds compared to modern ones. Besides mRNA expression, differential expression of non-coding RNA has been demonstrated to regulate adipogenesis and lipogenesis, although more research of different breeds in the transcriptional regulation is needed for better understanding of the underlying mechanisms that affect fatty phenotype.

## 5. Proteomic profile in local and modern pig breeds

Compared to relatively numerous transcriptomics studies, proteomic studies on subcutaneous adipose tissue and IMF comparing local and modern pig breeds are still relatively scarce (Table 5). The proteomic approach is, however, also important, as the abundance of a certain protein is not necessarily a reflection of its gene expression due to numerous post-transcriptional, translational and protein degradation changes [104].

### 5.1. Proteomic Profile Associated with Fat Metabolism in Subcutaneous Adipose Tissue

To the best of our knowledge, there has been only one study published comparing subcutaneous adipose tissue proteome between local and modern pig breeds. By comparing juvenile Basque and Large White pigs [105], several proteins were identified that were involved in the metabolic cycle of triglycerides. Pigs of the Basque breed exhibited a higher abundance of albumin, which suggests the presence of an extensive capillary network and a consequently higher capacity for fatty acid transport from blood to adipocytes. Moreover, lipogenic enzymes and lipolytic enzyme carboxylesterase 1 were also more abundant in the Basque pig breed, as well as proteins involved in acute phase response and low-grade inflammation. Additionally, the larger abundance of selenium-binding protein, which is essential for its reactive oxygen species scavenging properties, suggested increased oxidative stress related to adipocyte differentiation [105].

### 5.2. Proteomic Profile Associated with Fat Metabolism in Intramuscular Fat

Considering muscle tissue, the studies investigating the proteome profile and its association with IMF deposition between local and modern pig breeds are also quite rare, and the results are not always consistent. Comparisons of local Chinese Lantang and modern Landrace breed revealed a higher abundance of proteins related to oxidative metabolism and higher IMF content (pyruvate dehydrogenase) in fatty Lantang pigs, whereas leaner Landrace was characterized by a higher abundance of β-enolase, suggesting more glycolytic metabolism. Overexpression of albumin in Lantang pigs indicated a higher fatty acid transport capacity [106]. Similarly, a study conducted by Park et al. [50] revealed a higher abundance of albumin and fatty acid binding protein in native Korean than modern Landrace pigs. In contrast, a transcriptomic and proteomic study showed a higher abundance of proteins involved in glycolysis and glycolysis-related pathways for local Casertana than for Large White pigs [107].

### 5.3. Summary of Proteomic Profile Differences in Local and Modern Pig Breeds

The comparison of proteomic profiles between local and modern pig breeds confirmed the transcriptomic approach, thereby revealing several functional networks. In particular, a higher abundance of proteins involved in lipogenesis, fatty acid transport, lipolysis and immune response were demonstrated in local compared to modern pig breeds.

## 6. Adaptation and Selection Induced Specificities of Fatty Pigs

In Europe, the development of phenotypically distinct pig breeds chiefly occurred with the commencement of organized breeding in the 18^th^ century. Certain pig breeds were further submitted to intense selection pressure for lean meat content [108] and are nowadays used in intensive production systems. Many other European local breeds were abandoned (some are even extinct), and are today used on a limited scale. A common characteristic of these breeds is high adipogenic potential and low muscle mass deposition [109]. To better understand the genetic basis for the phenotypic differentiation, studies have focused on detection of selection signatures in order to detect possible candidate genes, and subsequently allelic differences between local and modern pig breeds [110,111,112]. 

With regard to differences in fat deposition, several comparative studies revealing regions that could be under adaptation or selection in fatty or lean pig breeds will be briefly described. A comprehensive study was performed by Herrero-Medrano et al. [111] in order to identify potential non-synonymous candidate mutations, which are responsible for phenotypic divergence between different local (i.e., Mangalitsa, Calabrese, Nera Siciliana, Casertana, Cinta Senese, Iberian, Chato Murciano, Tamworth, Middle white, Large black, Gloucester old spots, British Saddleback) and modern cosmopolitan pig breeds (i.e., Duroc, Piétrain, Large White, Landrace). The candidate genes with genomic variation in local breeds that may be under selection pressure and adaptation on different environments, were zinc-alpha-2-glycoprotein (stimulation lipid degradation in adipocytes) in Mangalica and Cinta Senese breeds, the taste 2 receptor member 40 for bitter taste as an adaptation to specific dietary environments in local compared to commercial breeds, and genes involved in immune response (for instance interleukin 12 receptor subunit beta 2, stabilin 1). All of these genes had previously been associated with backfat thickness and IMF [111]. Comparing local Chinese and modern cosmopolitan pigs also revealed regions which showed evidence of adaptation of the immune response in Chinese breeds (for instance the region with the Janus kinase 2 gene) [113]. Although the study of Muñoz et al. [114,115] only considered local pig breeds, it revealed several interesting candidate mutations with effects on fat deposition (polymorphisms in leptin receptor, melanocortin receptor 4) and composition (polymorphisms in SCD) which were segregating in many European local pig breeds [114]. Further study identified putative signals of selection for regions that may contain genes involved in fatness traits of the local breeds [115]. Candidate genes associated with higher fatness were genes involved in fatty acids metabolism (carnitine palmitoyl transferase I and acyl-CoA oxidase 1), genes associated with backfat thickness (NFKB inhibitor alpha, PPARG coactivator 1 beta), and gene (lipin-1) associated with obese pig phenotype, in Basque, Krškopolje and Cinta Senese, and Turopolje breeds, respectively. Some intermediates of the insulin signaling pathway were also observed, such as members of insulin-like growth factor binding protein gene family in Black Slavonian or insulin receptor substrate 1 in Casertana breeds [115].

## 7. Conclusions

Local pig breeds accumulate larger amounts of fat than modern ones, usually with higher proportions of saturated and monounsaturated than polyunsaturated fatty acids. Adipose tissue of local pig breeds exhibits a higher capacity for adipocyte hypertrophy and hyperplasia. These characteristics can be explained by higher de novo fatty acid synthesis, enhanced adipogenesis, and distinct lipid mobilization in local pig breeds compared to modern pig breeds. Studies involving transcriptomic and proteomic analysis of subcutaneous fat tissue reveal several groups of genes which differ between local and modern pig breeds. Local breeds exhibited upregulation of genes and proteins involved in lipogenesis, desaturation, immune response and fatty acid transport, while modern pig breeds exhibited upregulation of genes involved in extracellular matrix development and mitochondrial energy metabolism in subcutaneous adipose tissue. In the case of intramuscular fat, transcriptomic and proteomic studies indicate upregulation of genes and proteins involved in adipogenesis, lipogenesis, desaturation, lipolysis, fatty acid transport and immune response in local compared to modern pig breeds. Differences between fatty and lean pig breeds are mainly a result of selective breeding of modern pig breeds for fast growth and high leanness. However, several selection signatures were detected (e.g., regulation of fatty acid deposition, lipolysis and immune response) as possible genomic regions associated with specific fatty phenotypes in local pig breeds.

## Figures and Tables

**Table 1:** The function of lipogenic and lipolytic enzymes in adipose tissue [32].

Lipogenic Enzyme	Function
Acetyl-CoA carboxylase (ACACA)	Irreversible formation of malonyl-CoA from acetyl-CoA.
Fatty acid synthase (FAS)	Synthesis of palmitate from acetyl-CoA and malonyl-CoA.
Glucose-6-phosphate DH (G6PDH)	Providing NADPH for reductive biosynthesis of fatty acids.
Malic enzyme (ME)	Providing NADPH for reductive biosynthesis of fatty acids.
Stearoyl-CoA desaturase (SCD)	Transformation of MUFA from SFA.
Hormone-sensitive lipase (HSL)	Hydrolyses triglycerides to free fatty acids.
Lipoprotein lipase (LPL)	Catalyses the hydrolysis of triglycerides from circulating chylomicrons and very low-density lipoproteins.

DH = dehydrogenase; NADPH = dihydro-nicotinamide-adenine dinucleotide phosphate; MUFA = monounsaturated fatty acids; SFA = saturated fatty acids.

**Table 2:** Lipogenic enzyme activities in subcutaneous adipose tissue in local compared to modern pig breeds.

Ref.	Breed	Enzyme activities				
		ACACA	FAS	G6PDH	ME	SCD
[23]	Basque vs. LW	/	↓ *	/	↓ *	/
[34]	Alentejano vs. LW	↑2.7–3.7 FC	/	↑3.1–5.8 FC	↑6.2–6.9 FC	/
[35]	Ib vs. LNxLW	/	↑1.1–1.9 FC	↑1.2 FC	↑1.4–1.5 FC	/
[36]	Meishan vs. LW	/	/	/	/	↓1.4–1.8 FC

Ref. = reference; ACACA = acetyl-CoA carboxylase; FAS = fatty acid synthase; G6PDH = glucose-6-phosphate dehydrogenase; ME = malic enzyme; SCD = stearoyl-CoA-desaturase; LW = Large White; Ib = Iberian; LNxLW = Landrace x Large White; FC = fold change; ↑ higher enzyme activity in local pig breed compared to modern pig breed; ↓ lower enzyme activity in local pig breed compared to modern pig breed; / = activity not measured; * exact enzyme activities were not given, thus calculation of FC was not possible.

**Table 3:** Lipogenic enzyme activities in intramuscular fat for different muscles in local pig breeds compared to modern pig breeds.

Ref.	Breed	Enzyme Activities							
		Tissue	ACA	FAS	G6PDH	ME	SCD	LPL	HSL
[35]	Ib vs. LNxLW	LD	/	↑2.4 FC	ns	ns	/	/	/
[35]	Ib vs. LNxLW	BF	/	ns	ns	↑1.2–2.1 FC	/	/	/
[55]	Wujin vs. LN	LD	/	↑1.9 FC	/	/	↑2.1 FC	/	↓3.5 FC
[57]	Bas vs. LW	SM	↑ 1.4 FC	/	↑2.9 FC	↑1.8 FC	/	/	/
[58]	Ms vs. LW	SM	↑ *	/	↑ *	↑ *	/	/	/
[59]	Mas vs. LW	LD	ns	↑ *	/	/	/	ns	↓ *

Ref. = reference; ACA = acetyl-CoA carboxylase; FAS = fatty acid synthase; G6PDH = glucose-6-phosphate dehydrogenase; ME = malic enzyme; SCD = stearoyl-CoA desaturase; LPL = lipoprotein lipase; HSL = hormone sensitive lipase; Ib = Iberian; LN = Landrace; LW = Large White; LD = *longissimus dorsi* muscle; BF = *biceps femoris* muscle; SM = *semimembranosus* muscle; Bas = Basque; Ms = Meishan; Mas = Mashen; FC = fold change; ↑ higher enzyme activity in a local pig breed compared to a modern pig breed; ↓ lower enzyme activity in a local pig breed compared to a modern pig breed; ns = no statistically significant differences; / = not measured; * exact enzyme activities were not given, thus calculation of FC was not possible.

**Table 4:** Summary of comparative transcriptomic studies with the main findings between local and modern pig breeds associated with fat deposition.

Metabolic Pathway	Breed	Tissue	Platform	Ref.
**Adipocyte growth and lipid deposition**				
- ↑ lipogenesis, desaturation (ME1, ELOVL6, SCD)	Ib vs. Du	SCAT	qPCR	[38]
- ↑ lipogenesis, desaturation (FASN, SREBP-1, SCD)	Wujin vs. LN	LD-IMF	qPCR	[55]
- ↑ lipogenesis, desaturation (FASN, SCD)	DSP and Tibetan vs. LN and YY	LD-IMF	RNA-seq	[91]
- ↑ lipogenesis (ACACB)	Basque vs. LW	LD-IMF	microarray	[82]
- ↑ lipogenesis, desaturation (ELOVL6, ME1, PTGES3, AGPAT5, GNPAT, SCD)	Ib vs. Ib x Du	LD-IMF	microarray, qPCR	[83]
- ↑ lipogenesis (PCK1, FASN), desaturation (↑ SCD expression pigs at day 30, ↓ SCD expression pigs at day 150)	Jinhua vs. LN	LD-IMF	microarray	[92]
- ↑ lipogenesis (ME1, PCK1)	Ib vs. Du	SCAT	RNA-seq	[86]
- ↑ lipogenic and adipogenic gene expression after insulin and glucose exposure	Bamei vs. LW	SCAT, LD-IMF	qPCR	[84]
- ↑ adipogenesis (C/EBPγ, C/EBPα, PPARγ), lipogenesis (FASN)	Mashen vs. LN	LD-IMF	qPCR	[59]
- ↑ lipogenesis (e.g., PCK1, ACACB)	Songliao vs. LN	SCAT	RNA-seq	[88]
- ↑ adipogenic genes expression in preadipocyte cell culture in early stage of differentiation (PPARγ, CEBPα), ↑ lipogenic gene expression in late stage of differentiation (SREBP1, FASN)	Wujin vs. LN	LD-IMF	qPCR	[93]
**Lipid mobilization and expenditure**				
- ↓ lipolysis (HSL, ATGL)	Wujin vs. LN	LD-IMF	qPCR	[55]
- ↑ lipolysis (PON, PLA1A)	Ib vs. Ib x Du	LD-IMF	microarray	[83]
- ↑ lipolysis, fatty acid transport (LPL, LIPE, FABP3)	Jinhua vs. LN	LD-IMF	microarray	[92]
- ↑ lipolysis, fatty acid transport, oxidation (PPAP2A, LIPE, FABP3, SLC25A20, PPARδ)	Basque vs. LW	LD-IMF	microarray	[82]
- ↑ fatty acid transport, oxidation (FABP3, FABP4, CPT-1B)	Wujin vs. LN	LD-IMF	qPCR	[55]
- ↓ oxidoreductase activity, fatty acid degradation, mitochondrial function (e.g., ACAD, HADHA, ACAA2, HSD17B4)	Min vs. LN	SCAT	RNA-seq	[90]
- ↑ oxidoreductase activity	Chinese breeds * vs. YY	SCAT, LD-IMF	RNA-seq	[89]
- ↓ mitochondrial energy metabolism (e.g., SIRT3)	Basque vs. LW	SCAT	microarray	[72]
**Regulation**				
- ↑ response to steroid hormone stimulus	DSP and Tibetan vs. LN and YY	LD-IMF	RNA-seq	[91]
- ↑ LEP	Ib vs. Du	SCAT	qPCR	[38]
- ↑ LEP	Ib vs. Du	SCAT	RNA-seq	[86]
- ↑ insulin signaling pathway, insulin resistance	Songliao vs. LN	SCAT	RNA-seq	[88]
**Other**				
- ↑ immune response (e.g., CSF1R)	Basque vs. LW	SCAT	microarray	[72]
- ↑ immune response	Chinese breeds * vs. YY	SCAT, LD-IMF	RNA-seq	[89]
- ↑ immune response, ↓ extracellular matrix formation, ↓ growth, ↑ carbohydrate metabolism	Ib vs. Du	SCAT	RNA-seq	[86]
- ↑ glycolysis, ↑ gluconeogenesis	Songliao vs. LN	SCAT	RNA-seq	[88]

Ref. = references; ↑ = upregulation in local pig breeds compared to modern pig breeds; ↓ = downregulation in local pig breeds compared to modern pig breeds; ME1 = malic enzyme; ELOVL6 = elongation of very long chain fatty acids protein 6; SCD = stearoyl CoA desaturase; Ib = Iberian; Du = Duroc; SCAT = subcutaneous adipose tissue; qPCR = quantitative polymerase chain reaction; FASN = fatty acid synthase; SREBP-1 = sterol regulatory element-binding protein 1; LN = Landrace; LD-IMF = intramuscular fat of *longissimus dorsi* muscle; DSP = Diannan Small-ear pig; RNA-seq = RNA sequencing; YY = Yorkshire pig; ACACB = acetyl CoA carboxylase beta; LW = Large White; PTGES3 = prostaglandin E synthase 3; AGPAT5 = 1-acylglycerol-3-phosphate O-acyltransferase 5; GNPAT = glyceronephosphate O-acyltransferase, Ib x Du = Iberian x Duroc; PCK1 = phosphoenolpyruvate carboxykinase 1; C/EBPγ = CCAAT/enhancer-binding protein gamma; C/EBPα = CCAAT/enhancer-binding protein alpha; PPARγ = peroxisome proliferator-activated receptor gamma; HSL = hormone-sensitive lipase; ATGL = adipose triglyceride lipase; PON = paraoxonase; PLA1A = phospholipase A1 member A; LPL = lipoprotein lipase; LIPE = lipase E; FABP3 = fatty acid binding protein 3; PPAP2A = prostaglandin E synthase 3; SLC25A20 = solute carrier family 25 member 20; PPARδ = peroxisome proliferator activated receptor delta; FABP4 = fatty acid binding protein 4; CPT-1B = carnitine palmitoyl transferase 1B; ACAD = acyl-CoA dehydrogenase; HADHA = hydroxyacyl-CoA dehydrogenase; ACAA2 = acetyl-CoA acyltransferase 2; HSD17B4 = hydroxysteroid 17-beta dehydrogenase 4; SIRT3 = NAD-dependent deacetylase sirtuin-3; LEP = leptin; CSF1R = colony-stimulating factor 1 receptor; * Chinese breeds = Lantang, Tongcheng, Tibetan, Wuzhishan, Rongchang, Chenghua and Neijiang.

**Table 5:** Summary of comparative proteomics studies with the main findings between local and modern pig breeds associated with fat deposition.

Metabolic pathway	Breed	Tissue	Ref.
**Lipid deposition**			
↑ lipogenesis (ME, G6PDH)	Basque vs. LW	SCAT	[105]
**Lipid mobilization and expenditure**			
↑ fatty acid transport capacity (albumin, fatty acid binding protein)	Korean vs. LN	LD-IMF	[50]
↑ fatty acid transport capacity (albumin), ↑ lipolysis (CES)	Basque vs. LW	SCAT	[105]
↑ fatty acid transport capacity (albumin)	Lantang vs. LN	LD-IMF	[106]
**Other**			
↑ acute phase response (ITIH) and low-grade inflammation (serpins), ↑ selenium binding protein	Basque vs. LW	SCAT	[105]
↑ carbohydrate metabolism (pyruvate dehydrogenase), ↑ oxidative metabolism (COX5A, ATP5), ↓ glycolytic metabolism (β-enolase)	Lantang vs. LN	LD-IMF	[106]
↑ glycolysis and glycolysis-related pathways (β-enolase, TPI, PGM1, LDH, CK and GPDH)	Casertana vs. LW	LD-IMF	[107]

Ref. = reference; ↑ upregulation in local pig breeds compared to modern pig breeds; ↓ downregulation in local pig breeds compared to modern pig breeds; ME = malic enzyme; G6PDH = glucose-6-phosphate dehydrogenase; LW = Large White; SCAT = subcutaneous adipose tissue; LN = Landrace; LD-IMF = *longissimus dorsi* muscle intramuscular fat; CES = carboxylesterase; ITIH = inter-alpha-trypsin inhibitor-4; COX5A = cytochrome c oxidase subunit 5a; ATP5 = ATP synthase subunit 5; TPI = triosephosphate isomerase; PGM1 = phosphoglucomutase 1; LDH = lactate dehydrogenase; CK = creatine kinase; GPDH = glycerol-3-phosphate dehydrogenase.
